# Systemic Metastatic Progression of Canine Mammary Comedocarcinoma with Pathological Femoral Fracture: A Case Report

**DOI:** 10.3390/vetsci13101047

**Published:** 2026-10-06

**Authors:** Jihyun Moon, Sangyul Lee, Ki-Dong Eom, Hwi-Yool Kim, Jung-Moon Kim

**Affiliations:** 1 Department of Veterinary Surgery, College of Veterinary Medicine, Konkuk University, Neungdong-ro 120, Seoul 05029, Republic of Korea; kumelody812@konkuk.ac.kr (J.M.); sangyul7918@gmail.com (S.L.); hykim@konkuk.ac.kr (H.-Y.K.); 2 Department of Veterinary Medical Imaging, College of Veterinary Medicine, Konkuk University, 120 Neungdong-ro, Gwangjin-gu, Seoul 05029, Republic of Korea; eomkd@konkuk.ac.kr

**Keywords:** canine mammary carcinoma, comedocarcinoma, skeletal metastasis, pathological fracture, distant metastasis

## Abstract

Canine mammary comedocarcinoma is considered an aggressive histological subtype with a high potential for metastatic dissemination. A 13-year-old intact female Jindo-mixed dog underwent mastectomy for a grade II mammary comedocarcinoma, and no metastatic involvement was identified in the regional lymph nodes or thorax at initial staging. On postoperative day (POD) 65, the dog developed an atraumatic pathological fracture of the left femoral diaphysis. Despite two surgical fixation procedures, the fracture progressed to non-union with progressive osteolysis. During subsequent follow-up, a subcutaneous mass and radiographic evidence of pulmonary metastasis were identified, and metastatic involvement of the femur was subsequently supported by histopathological examination. The dog died spontaneously on POD 188 following progressive disease and deterioration in clinical condition. Necropsy revealed extensive metastatic carcinoma involving the femur, medial iliac lymph node, lungs, and surrounding soft tissues. This case describes rapid and extensive multisystemic metastatic progression of canine mammary comedocarcinoma despite initially negative regional lymph node and thoracic staging. Notably, the atraumatic pathological femoral fracture was the first clinically recognized manifestation of metastatic disease. This case highlights the potential for skeletal involvement to cause substantial morbidity during metastatic progression of canine mammary carcinoma and emphasizes the importance of considering skeletal metastasis in dogs with aggressive mammary carcinoma presenting with unexplained pathological fractures or persistent failure of fracture healing.

## Simple Summary

A 13-year-old dog underwent surgical removal of a mammary tumor diagnosed histologically as grade II comedocarcinoma. Sixty-five days after surgery, the dog developed an atraumatic pathological fracture of the femur, which failed to heal despite two surgical repairs. During subsequent follow-up, metastatic disease involving the femur, subcutaneous tissue, medial iliac lymph node, and lungs was identified, and necropsy confirmed widespread metastatic dissemination. The dog subsequently died spontaneously on postoperative day (POD) 188 following progressive disease and deterioration in clinical condition. This case highlights an uncommon presentation of canine mammary carcinoma in which a pathological fracture was the first clinically recognized manifestation of metastatic disease. Although skeletal metastasis is considered uncommon in dogs with mammary carcinoma, it should be included among the differential diagnoses for unexplained pathological fractures in dogs with aggressive mammary tumors, even when other metastatic lesions are not initially evident.

## 1. Introduction

Canine mammary tumors (CMTs) are among the most common neoplasms in female dogs and comprise a heterogeneous group of tumors with diverse biological behaviors and clinical outcomes [1,2,3,4]. Histopathological subtype, tumor grade, tumor size, and lymphatic or vascular invasion have been associated with tumor progression, metastatic potential, and survival [1,5]. Among malignant mammary tumors, comedocarcinoma is characterized histologically by solid aggregates of neoplastic epithelial cells with prominent central necrosis and has been associated with aggressive biological behavior and a poor prognosis [6,7,8,9]. These histopathological characteristics may contribute to local invasion and metastatic dissemination [1].

Metastatic disease is a major determinant of outcome in dogs with malignant mammary tumors. Canine mammary carcinomas can disseminate through lymphatic and hematogenous routes, with regional lymph nodes and lungs being the most commonly recognized sites of metastasis [2,10]. Metastatic lesions may also occur in less frequently affected organs, including the liver, spleen, and skeletal system [2,10,11]. The distribution of metastatic lesions can vary among individual dogs, and multisystemic dissemination may develop during the course of progressive disease.

Skeletal metastasis is considered uncommon in dogs with malignant neoplasia but may be underrecognized in clinical practice [12,13]. Skeletal metastases have been reported in both the axial and appendicular skeleton, including the vertebrae, humerus, and femur [11,14,15]. The apparent low frequency of skeletal metastasis may partly reflect limitations in clinical detection, as skeletal imaging is not routinely performed in dogs without musculoskeletal abnormalities. Consequently, some skeletal lesions may remain clinically occult until they produce pain, lameness, bone destruction, or pathological fracture. Clinically significant skeletal metastasis can result in progressive osteolysis, impaired bone integrity, and pathological fracture, substantially compromising quality of life [16,17].

Although skeletal metastasis has been documented in dogs with mammary carcinoma, detailed descriptions of its clinical progression and association with multisystemic metastatic disease remain limited [10,11,13,14,16]. In particular, longitudinal clinical and imaging findings associated with mammary carcinoma-related skeletal metastasis are poorly characterized. The development of a pathological fracture as an initial clinically recognized manifestation of metastatic mammary carcinoma may therefore present a diagnostic challenge, particularly when overt metastatic lesions are not detected during the initial staging evaluation.

In this report, we describe the longitudinal clinical, radiographic, cytological, histopathological, and postmortem findings of a dog with grade II mammary comedocarcinoma that developed progressive metastatic disease involving the femur, subcutaneous tissue, lymph nodes, and lungs. Skeletal involvement was first clinically recognized as an atraumatic pathological fracture of the femur, followed by progressive osteolysis, failure of fracture healing, and detection of additional metastatic lesions. This case provides a detailed description of an uncommon skeletal metastatic presentation of canine mammary comedocarcinoma and highlights the importance of considering skeletal metastasis in dogs with aggressive mammary carcinoma presenting with an unexplained pathological fracture.

## 2. Case Description

### 2.1. Initial Presentation, Diagnosis, and Staging

**Figure 1:**
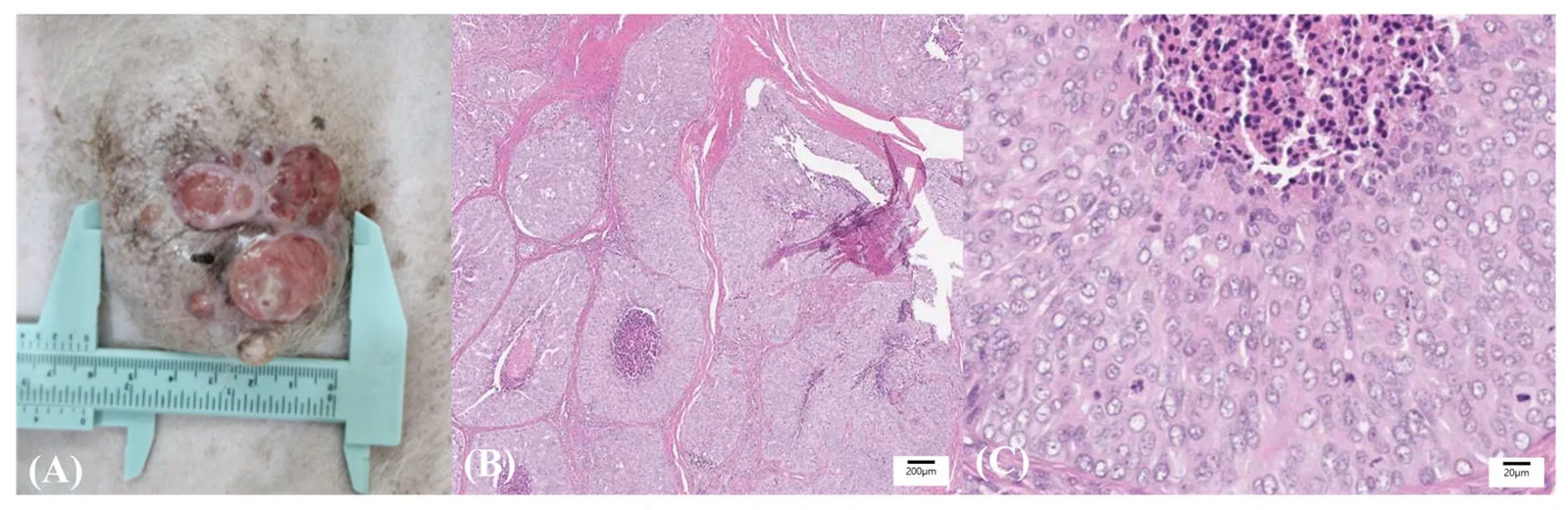
Gross and histopathological features of the primary left mammary tumor. (**A**) Gross appearance of the left mammary gland mass, showing multiple irregular, nodular, and ulcerated areas. (**B**) Histopathological examination showing variably sized neoplastic cell islands separated by fibrous connective tissue, with the neoplastic cells predominantly arranged in solid sheets. (**C**) Histopathological examination showing neoplastic cells with moderate to abundant eosinophilic cytoplasm, hyperchromatic nuclei, prominent nucleoli, and marked anisokaryosis. Hematoxylin and eosin stain.

A 13-year-old intact female Jindo-mixed dog weighing 20 kg was presented to Konkuk University Veterinary Hospital for evaluation of generalized weakness and a mammary gland mass. Physical examination revealed a large, firm, immobile mass involving the left fourth mammary gland. The mass had an irregular margin with multiple nodular and cystic areas and measured approximately 9 × 6 × 2 cm (length × width × height) (Figure 1). The overlying skin exhibited local inflammatory changes, including increased warmth, erythema, erosion, pain, and bleeding. At the initial presentation, complete blood count, serum biochemical analysis, and electrolyte analysis revealed no clinically significant abnormalities. Radiographic examination showed no definite evidence of metastatic disease, and abdominal ultrasonography revealed no findings suggestive of metastatic involvement.

Fine-needle aspiration (FNA) cytology of the mammary mass revealed a malignant epithelial neoplasm, and mammary carcinoma was suspected. On 8 August 2024, the dog underwent bilateral mastectomy, ovariohysterectomy, and bilateral superficial inguinal lymphadenectomy. The left-sided mastectomy included the first through fifth mammary glands, whereas the right-sided mastectomy included the fourth and fifth mammary glands. Preoperative thoracic radiographs showed no radiographic evidence of pulmonary metastasis.

Histopathological examination of the left fourth mammary gland mass confirmed mammary comedocarcinoma, histologic grade II, characterized by an infiltrative growth pattern and variably sized islands of neoplastic epithelial cells separated by abundant fibrous connective tissue. The neoplastic cells were predominantly arranged in solid sheets, with prominent central comedo-type necrosis. The cells had moderate to abundant eosinophilic cytoplasm and hyperchromatic nuclei with one to two prominent nucleoli. Marked anisokaryosis was observed, and 2–5 mitotic figures were identified per high-power field at 400× magnification. Tumor emboli were also observed. Multifocal severe necrosis, cartilage metaplasia, and squamous differentiation were present within the tumor, accompanied by multifocal infiltration of lymphocytes and plasma cells. Ulceration of the overlying epidermis was also observed (Figure 1). Both excised superficial inguinal lymph nodes were negative for metastatic involvement on histopathological examination. Based on the primary tumor size (>5 cm; T3), histologically negative superficial inguinal lymph nodes (N0), and no detectable distant metastatic disease on initial staging examinations (M0), the tumor was classified as clinical stage III (T3N0M0) according to the modified TNM staging system for canine mammary tumors [11,18,19,20].

### 2.2. Postoperative Course

The postoperative period was complicated by surgical-site dehiscence and skin flap ischemia, requiring revision surgery. The wound was subsequently managed by second-intention healing. No further major surgical-site complications were documented during follow-up before the development of the femoral lesion.

### 2.3. Development of Pathological Femoral Fracture

**Figure 2:**
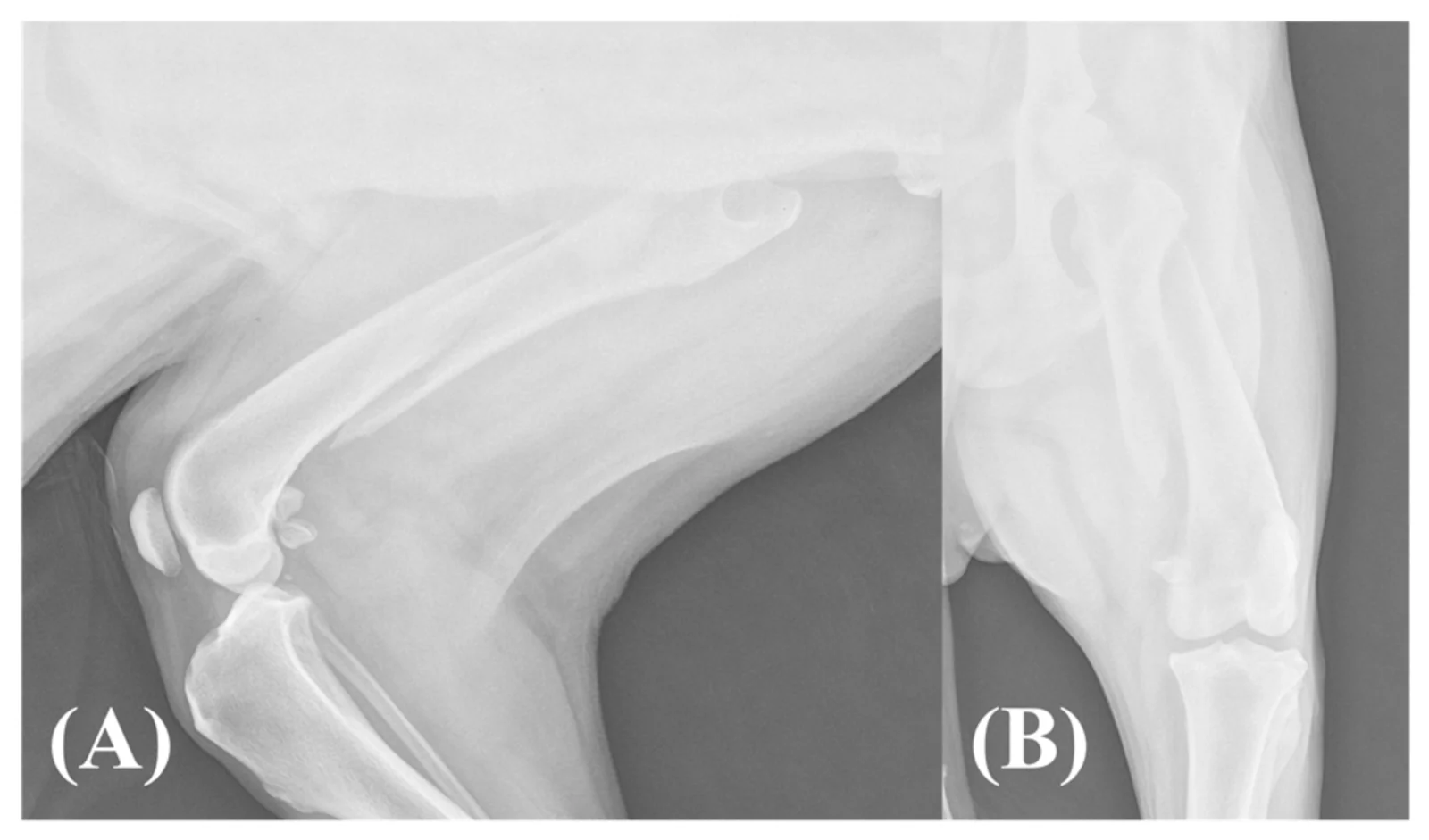
Radiographic findings of a long-oblique fracture of the left femoral diaphysis: (**A**) mediolateral view; (**B**) anteroposterior view.

On postoperative day (POD) 65, the dog developed acute left pelvic limb lameness without a known history of trauma. Orthopedic radiographs revealed a long-oblique fracture of the left femoral diaphysis (Figure 2). Given the recent diagnosis of mammary comedocarcinoma, a pathological fracture associated with underlying skeletal disease, including possible metastatic involvement, was considered among the differential diagnoses. Thoracic radiographs obtained at that time showed no radiographic evidence of pulmonary metastatic disease.

**Figure 3:**
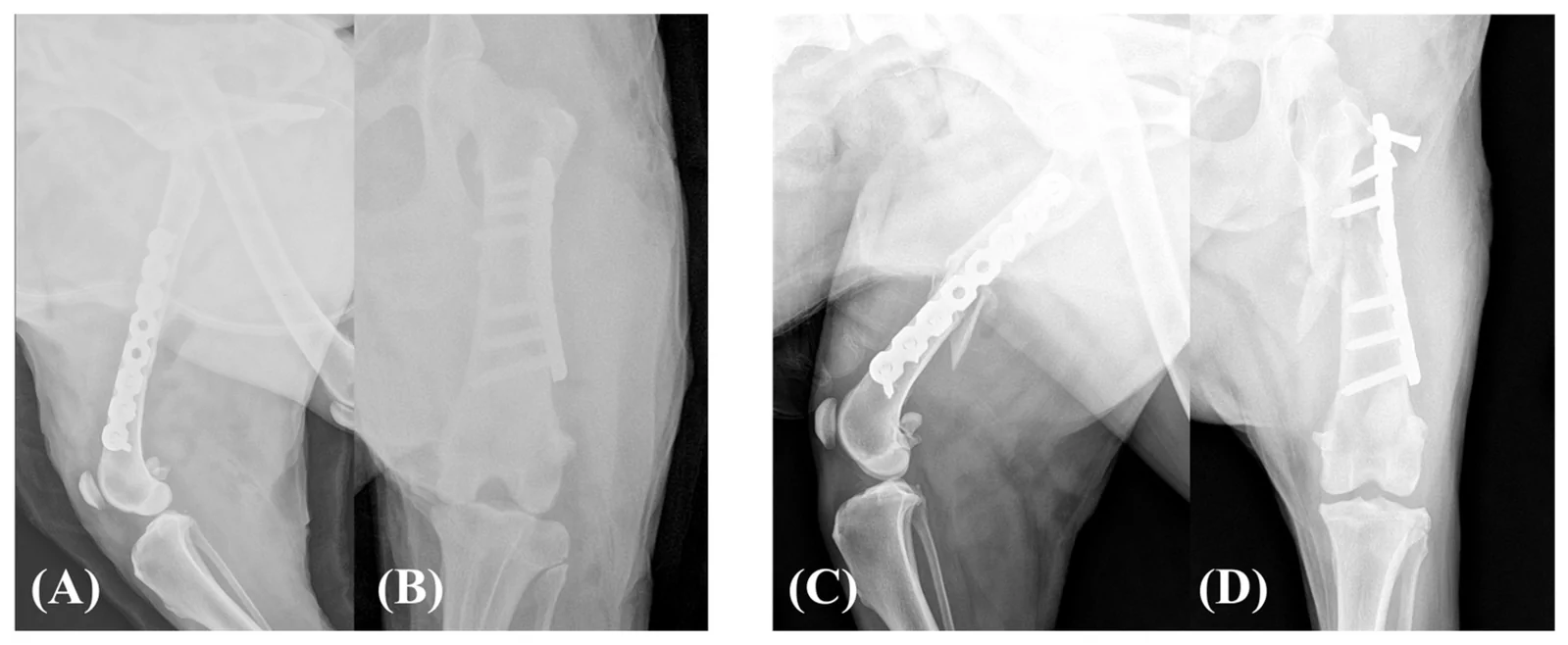
Postoperative radiographs of the left femur following internal fixation of the pathological fracture and subsequent refracture. (**A**,**B**) Mediolateral and anteroposterior radiographs obtained on POD68 after internal fixation with a bone plate and screws. (**C**,**D**) Mediolateral and anteroposterior radiographs obtained on POD82 demonstrating refracture of the left femur despite surgical stabilization.

**Figure 4:**
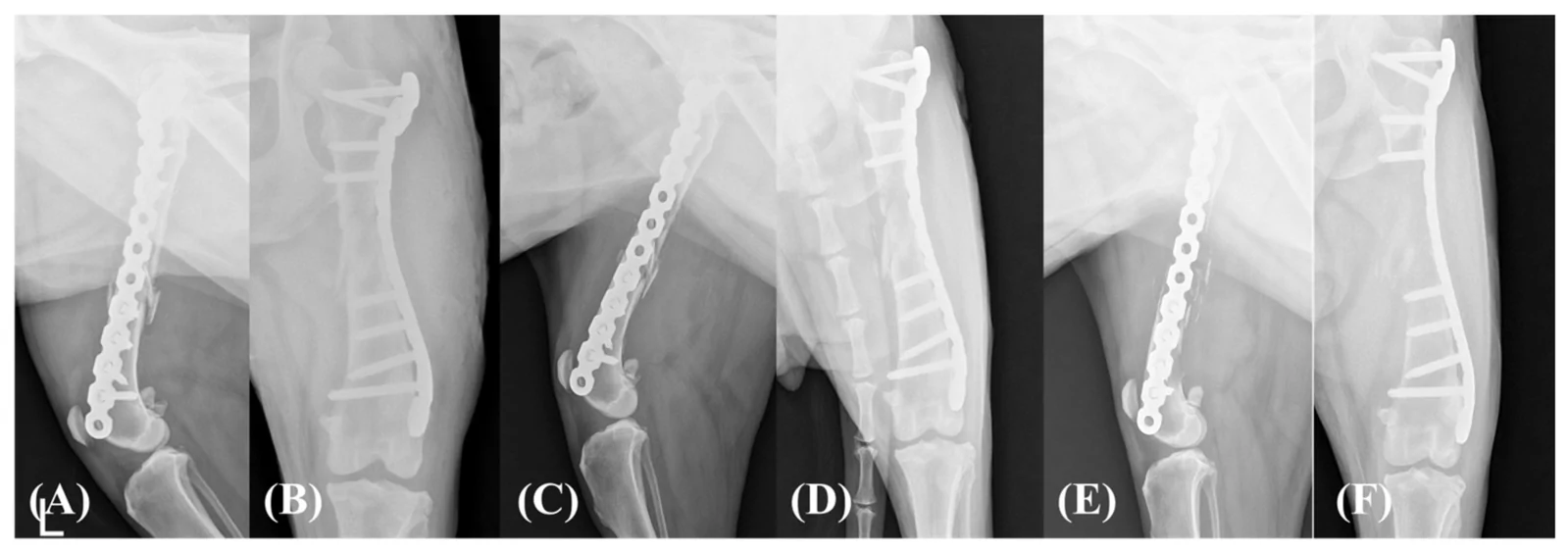
Postoperative radiographs of the left femur following revision fixation. (**A**) Mediolateral and (**B**) anteroposterior radiographic views showing stabilization of the femoral fracture with a bone plate and screws. Follow-up radiographs obtained on postoperative day (POD) 102 showing decreased cortical continuity at the proximal fracture margin with early evidence of osteolysis: (**C**) mediolateral and (**D**) anteroposterior views. Follow-up radiographs obtained on POD 113 demonstrating progression of the femoral lesion, characterized by diffuse reduction in femoral radiopacity, disruption of cortical continuity, decreased osseous apposition, and persistent fracture non-union: (**E**) mediolateral and (**F**) anteroposterior views. Progressive osteolysis and identification of multisite metastasis.

Internal fixation was performed on POD 68 using a bone plate and screws, with three screws placed in each of the proximal and distal fracture segments (Figure 3). Despite stabilization, the dog developed recurrent lameness, and refracture occurred on POD 82 (Figure 3). Compared with the initial postoperative radiographs, decreased radiopacity of the bone fragments surrounding the fracture site and reduced osseous apposition were observed from both the anteroposterior (AP) and mediolateral (ML) views. Thoracic radiographs obtained at the time of refracture again showed no radiographic evidence of pulmonary metastatic disease. Revision fixation was subsequently performed using a bone plate and screws, with four screws placed in each of the proximal and distal segments (Figure 4).

**Figure 5:**
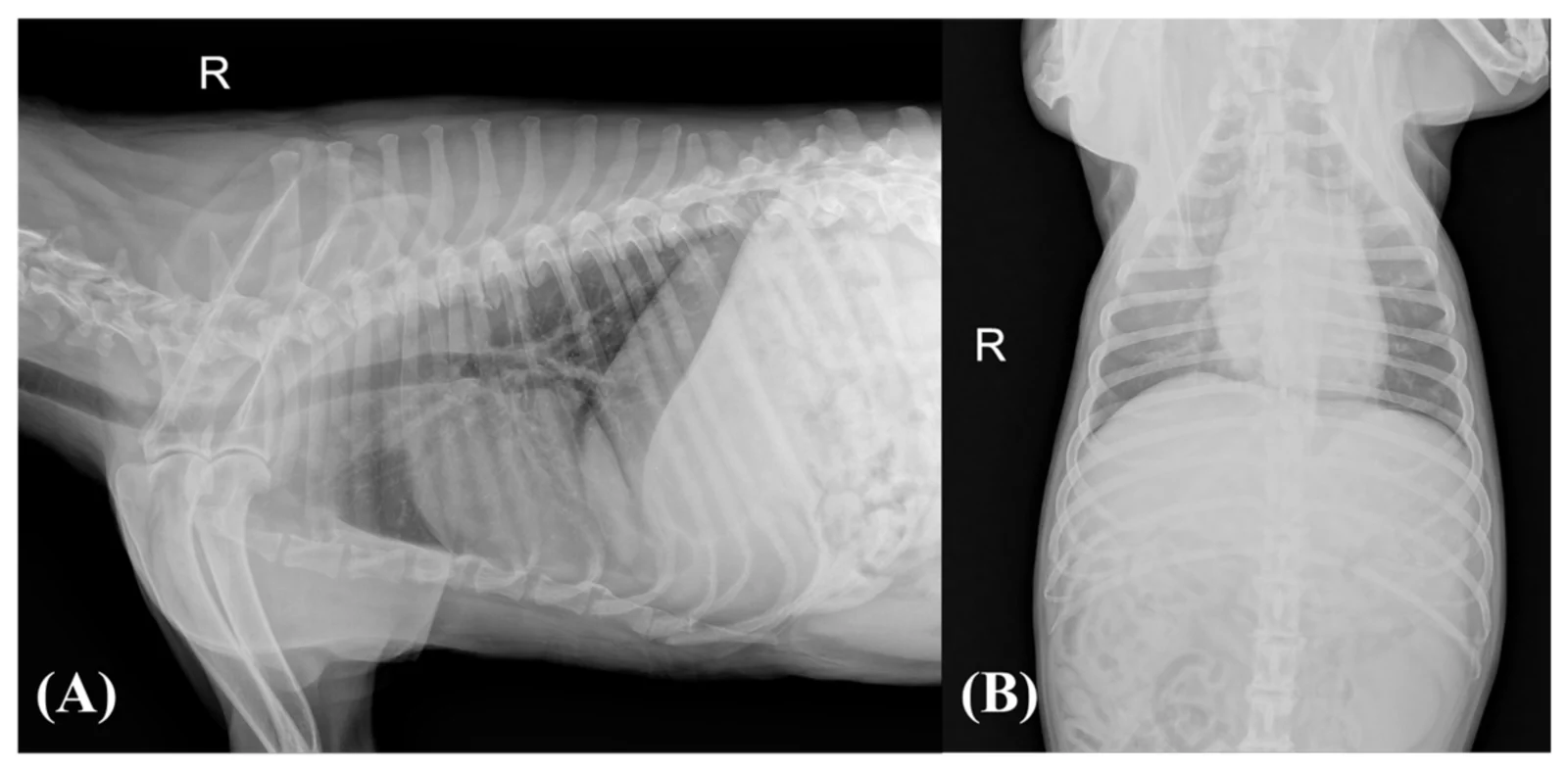
Thoracic radiographs obtained on postoperative day 102 revealing a round soft-tissue opacity in the right cranial lung lobe suspicious for pulmonary metastasis: (**A**) Right lateral view; (**B**) Ventrodorsal view. R, right side.

Follow-up radiographs obtained on POD 102 demonstrated decreased cortical continuity at the proximal fracture margin with early evidence of osteolysis (Figure 4). Thoracic radiographs obtained at the same time revealed a round soft-tissue opacity in the right cranial lung lobe, raising suspicion for pulmonary metastasis (Figure 5). By POD 113, the femoral lesion had progressed, characterized by diffuse reduction in femoral radiopacity, disruption of cortical continuity, reduced osseous apposition, and persistent fracture non-union despite repeated surgical stabilization (Figure 4).

**Figure 6:**
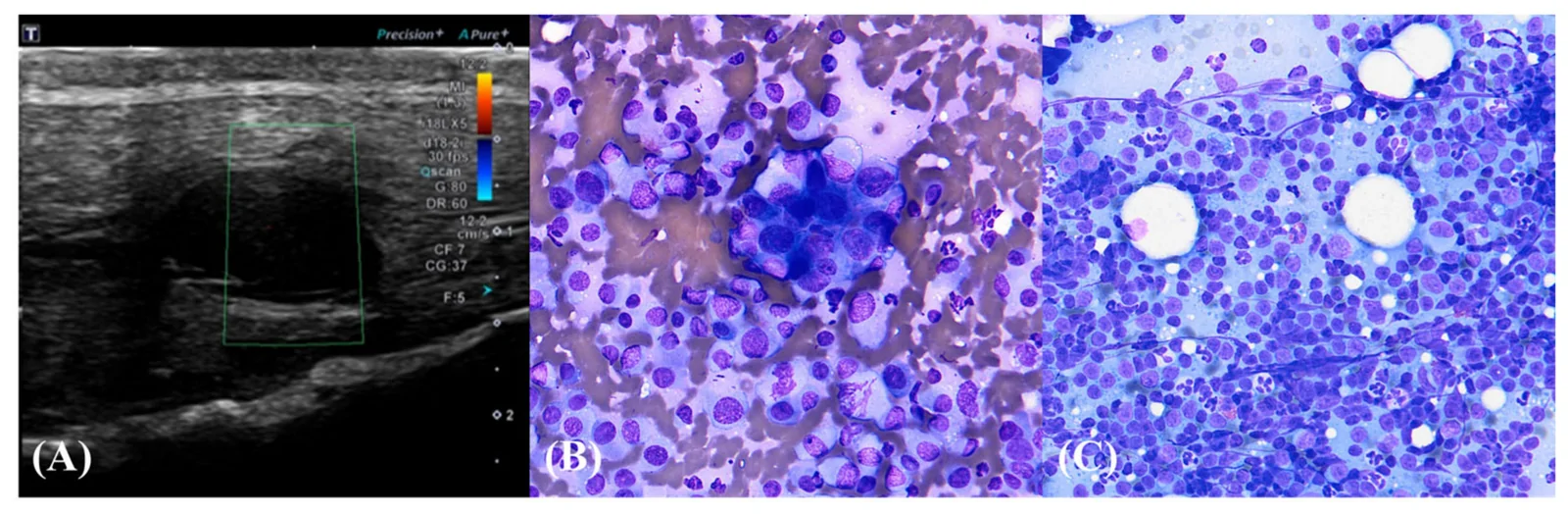
Diagnostic evaluation of the subcutaneous mass and left popliteal lymph node. (**A**) Ultrasonographic image of the subcutaneous mass in the left pelvic limb. (**B**) Cytology of the subcutaneous mass shows highly cellular cohesive clusters of neoplastic cells with marked anisokaryosis. (**C**) Cytology of the left popliteal lymph node demonstrating neutrophilic and eosinophilic lymphadenitis without definitive metastatic cells.

A subcutaneous mass identified in the left pelvic limb (laterocaudal area of the left femur) at this time was evaluated by FNA cytology (Figure 6). The aspirate contained a population of neoplastic epithelial cells, morphologically compatible with malignant carcinoma and highly suspicious for metastatic mammary carcinoma based on the patient’s history of comedocarcinoma. In contrast, cytological examination of the left popliteal lymph node revealed no definitive evidence of metastasis, displaying features consistent with neutrophilic and eosinophilic lymphadenitis secondary to regional inflammation (Figure 6).

**Figure 7:**
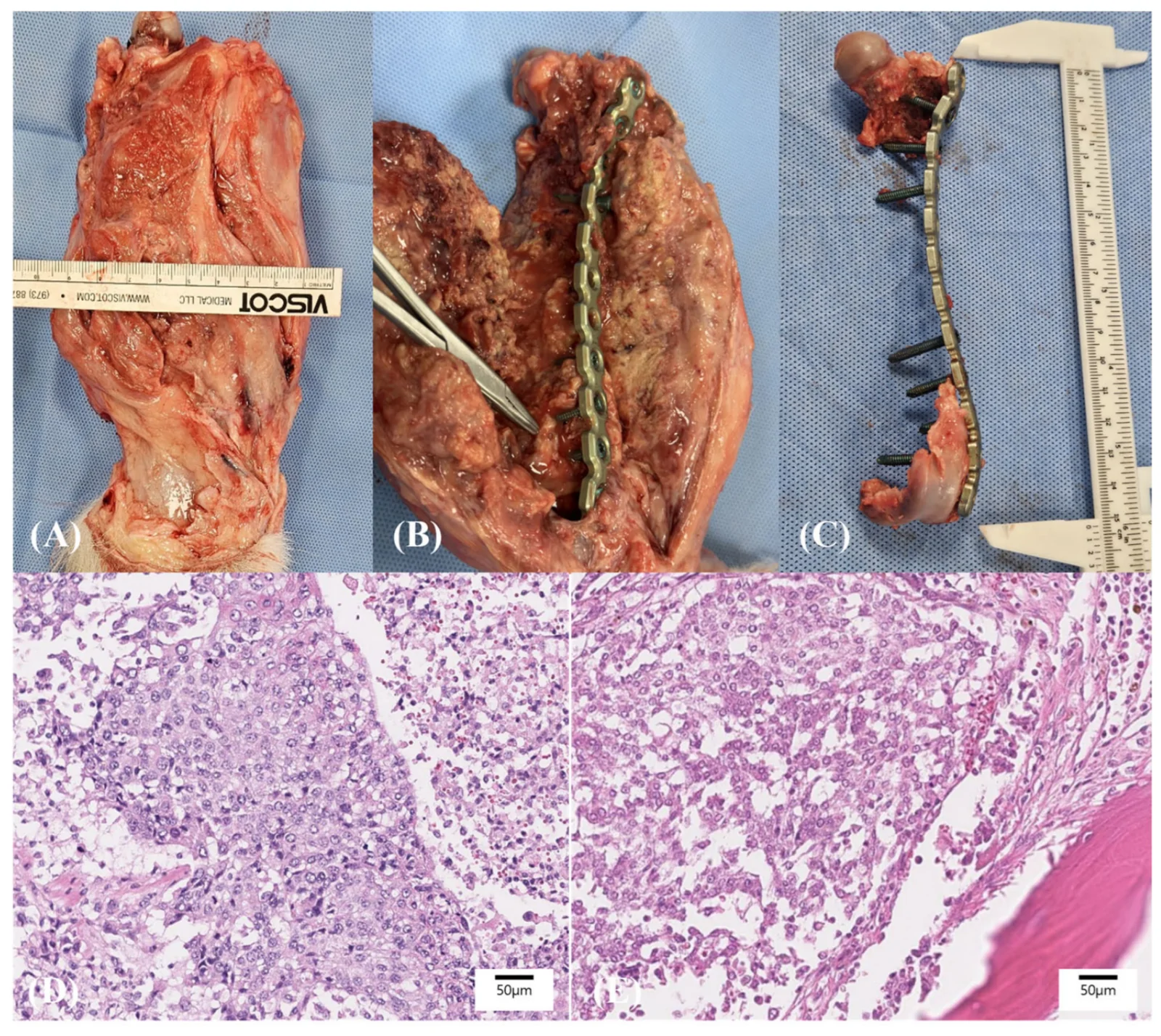
Gross and histopathological findings of the left pelvic limb and femur at necropsy: (**A**) Gross appearance of the large neoplastic mass occupying the left thigh; (**B**) Cut surface showing the internal structure of the tumor; (**C**) Gross appearance at necropsy of the remaining femur with the implant construct in situ; (**D**) Histopathological section of the soft tissue specimen showing cohesive aggregates of pleomorphic epithelial tumor cells with focal squamous differentiation, accompanied by severe necrosis and hemorrhage (H&E stain); (**E**) Histopathological section of the left femur showing an aggregate of neoplastic cells within the medullary cavity (H&E stain).

Given the progressive osteolysis, persistent fracture non-union, and concurrent identification of additional neoplastic lesions, metastatic involvement of the femur was strongly suspected. Biopsy of the affected femoral region demonstrated an epithelial neoplasm infiltrating the bone tissue, with morphological features compatible with those of the previously diagnosed mammary carcinoma (Figure 7). These findings supported metastatic mammary carcinoma involving the femur.

During this period, analgesic management consisted of multimodal therapy with gabapentin, firocoxib, and tramadol. Despite dose escalation, pain became increasingly difficult to control, accompanied by progressive deterioration in the dog’s functional status and quality of life. As the disease progressed, euthanasia was discussed with the owner as an option for end-of-life management. However, the owner elected to continue treatment and supportive care. The dog was not euthanized and died spontaneously on POD 188.

### 2.4. Necropsy Findings

The dog died spontaneously on POD 188 following progressive disease and deterioration in clinical condition. Necropsy revealed extensive neoplastic involvement of the left pelvic limb and femur. A large mass measuring approximately 9 × 15 × 5 cm occupied the left thigh and contained extensive areas of necrosis. Histopathological examination revealed an epithelial neoplasm in the soft tissue, with aggregates of neoplastic cells identified within the medullary cavity of the femur. The morphological features were compatible with metastatic mammary carcinoma (Figure 7).

**Figure 8:**
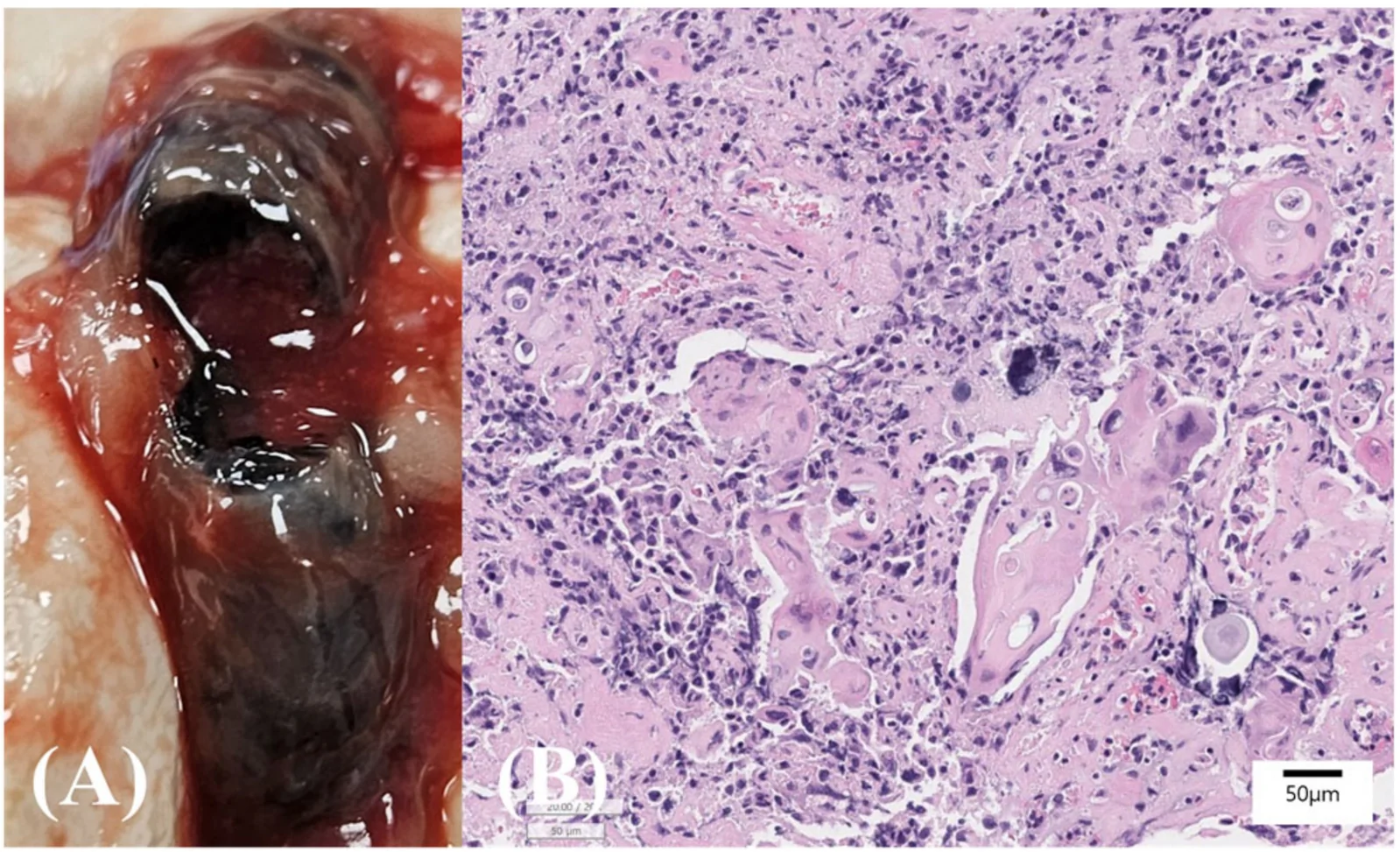
Left medial iliac lymph node: (**A**) Gross appearance of the enlarged left medial iliac lymph node showing internal ulceration due to neoplastic involvement; (**B**) Histopathological section showing neoplastic cells under high-power field (HPF, H&E stain).

**Figure 9:**
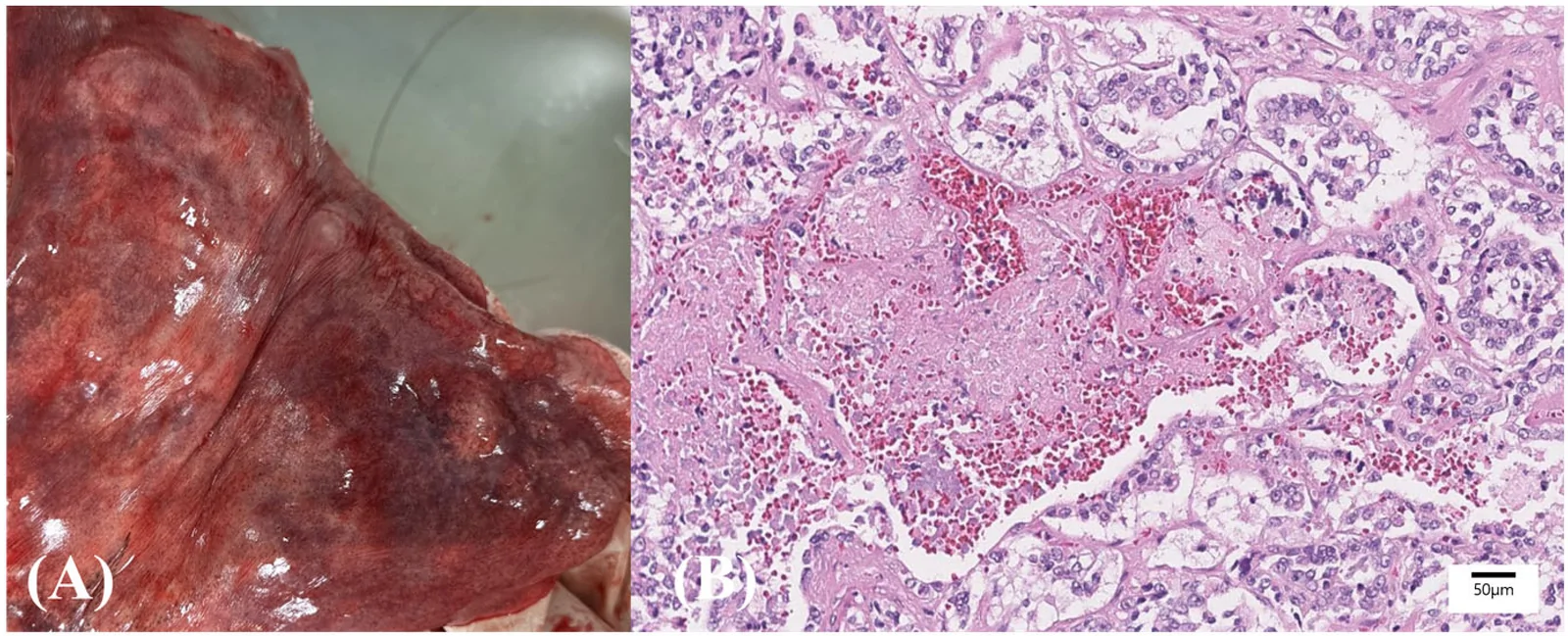
Multiple lung nodule metastasis suspected: (**A**) Multiple nodules scattered throughout all lung lobes; (**B**) High-power field (HPF) view of the lung nodule demonstrating mixed tubular and solid growth patterns with marked nuclear pleomorphism, intracytoplasmic vacuoles, and high mitotic activity (11/10 HPF, H&E stain).

The left medial iliac lymph node was enlarged, measuring 2.5 × 1.5 × 0.6 cm, and exhibited extensive neoplastic involvement and internal ulceration (Figure 8). Histopathological examination confirmed metastatic carcinoma. Based on the known primary mammary tumor and the morphological and distributional characteristics of the metastatic lesions, the metastatic carcinoma was considered most consistent with mammary origin. Multiple pulmonary nodules were identified throughout all lung lobes, and histopathological examination confirmed metastatic epithelial neoplasia, considered most consistent with metastasis from the primary mammary carcinoma (Figure 9).

Collectively, the clinical, radiographic, cytological, histopathological, and necropsy findings supported metastatic involvement of the femur, lungs, subcutaneous tissue, and regional lymph nodes in association with the previously diagnosed mammary comedocarcinoma. At initial staging, the tumor had been classified as clinical stage III based on its size and absence of detectable regional lymph node or distant metastasis [20,21,22]. The chronological sequence of metastatic development at individual sites could not be definitively established, as metastatic lesions may have been present before becoming clinically, radiographically, or cytologically detectable.

## 3. Discussion

Canine mammary carcinomas exhibit heterogeneous biological behavior, ranging from relatively indolent tumors to highly aggressive neoplasms with regional and distant metastasis. The lungs and regional lymph nodes are the most commonly recognized sites of metastasis, whereas dissemination to other organs is less frequently documented [11]. Previous studies have reported that distant metastasis occurs in a substantial proportion of dogs with invasive mammary carcinoma during follow-up, although the reported frequency may vary depending on the extent and method of staging [12,13,21,22]. Lymphovascular invasion, older age, multicentricity, and a high Ki-67 proliferation index have also been associated with shorter distant metastasis-free intervals [4,23]. These clinicopathological features may therefore help identify dogs at increased risk of systemic metastatic progression.

**Figure 10:**
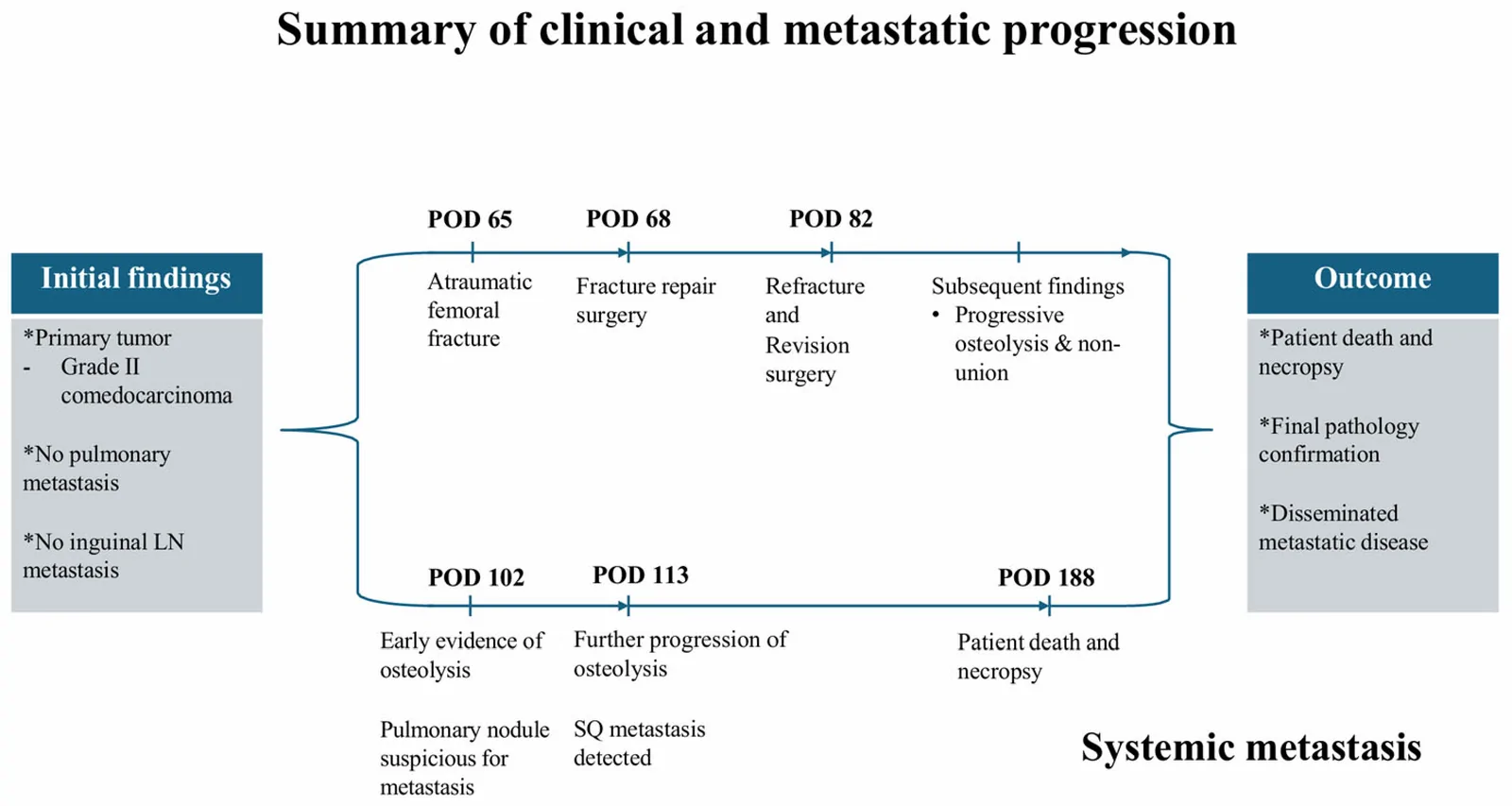
Schematic timeline outlining the clinical course and progression of disseminated metastatic disease (LN, lymph node; POD, postoperative day; SQ, subcutaneous).

The present case was characterized by extensive metastatic disease involving the subcutaneous tissue, regional lymph nodes, the lungs, and the skeletal system. No pulmonary or inguinal lymph node metastasis was identified at initial staging. The primary tumor showed aggressive histopathological features, including marked cellular atypia, tumor emboli, extensive necrosis, and infiltrative growth. The dog subsequently developed an atraumatic femoral fracture, followed by refracture after internal fixation. By POD 102, progressive osteolysis at the fracture site was evident radiographically, and a pulmonary nodule suspicious for metastasis was identified. By POD 113, the osteolysis had progressed further and persistent non-union was evident, while a subcutaneous metastatic lesion was clinically recognized (Figure 10).

Comedocarcinoma is considered an aggressive subtype of canine mammary carcinoma and has been associated with an unfavorable prognosis [8,21,24,25]. The extensive metastatic distribution observed in this dog is therefore consistent with the reported aggressive behavior of this subtype, although the specific biological factors underlying the metastatic phenotype could not be determined because molecular and proliferative profiling was not performed.

Skeletal metastasis represents an uncommon but clinically important manifestation of metastatic carcinoma in dogs [16,26,27]. Mammary gland tumors have been identified among the primary neoplasms associated with skeletal metastasis, with lesions reported in both the axial and appendicular skeleton [12,13,16,28]. Skeletal metastasis may remain clinically occult until sufficient bone destruction develops to cause pain, lameness, or pathological fracture [12,16,29].

In the present case, the pathological femoral fracture was the first clinically recognized manifestation suggesting metastatic progression beyond the primary mammary tumor. The fracture developed without a known history of trauma only 65 days after mastectomy and was followed by refracture, progressive osteolysis, and persistent non-union despite repeated internal fixation. Although pathological fractures can result from various primary or metastatic skeletal diseases, the combination of progressive osteolysis, failure of fracture healing, and subsequent identification of additional metastatic lesions strongly supported metastatic involvement of the femur in this dog. Therefore, in dogs with a known aggressive mammary carcinoma, an unexplained fracture or persistent failure of fracture healing should prompt consideration of underlying metastatic disease.

The biological mechanisms responsible for skeletal dissemination of canine mammary carcinoma remain poorly characterized. In human breast cancer, metastatic colonization of bone involves complex interactions between tumor cells and the bone microenvironment [30]. Tumor-derived factors can promote osteoclastic bone resorption and release growth factors stored within the bone matrix, thereby creating a microenvironment that supports further tumor growth and bone destruction [30,31,32]. Similar mechanisms may potentially contribute to skeletal metastasis in canine mammary carcinoma; however, direct extrapolation from human breast cancer is inappropriate in the absence of species-specific evidence. The present case was characterized by marked osteolysis and destruction of the femur, but the mechanisms underlying these changes could not be investigated because molecular profiling and immunohistochemical characterization were not performed.

The distribution of metastatic lesions in this dog also illustrates the complexity of metastatic progression. Regional lymph node involvement is consistent with lymphatic dissemination, whereas pulmonary and skeletal involvement may occur through hematogenous dissemination [11]. However, the identification of these lesions at different time points should not be interpreted as evidence of their biological sequence. In particular, the femoral fracture preceded radiographic detection of pulmonary metastasis, but this does not establish that skeletal metastasis occurred before pulmonary dissemination. Subclinical lesions may have been present before they became detectable by radiography, cytology, or clinical examination. Therefore, the most appropriate interpretation of this case is that the femoral fracture represented the first clinically recognized manifestation of metastatic disease, rather than necessarily the first site of metastatic colonization.

An additional diagnostic consideration in this case was distinguishing skeletal metastasis from a primary bone neoplasm. Metastatic carcinoma involving bone may mimic a primary skeletal neoplasm, particularly when extensive bone destruction is present [12,13]. In this dog, epithelial neoplastic cells were identified within the affected femoral tissue, and their morphological features were compatible with those of the previously diagnosed mammary comedocarcinoma. The presence of concurrent metastatic lesions in the subcutaneous tissue, lymph nodes, and lungs further supported a metastatic mammary origin. However, immunohistochemical confirmation was not performed. Therefore, another primary epithelial neoplasm cannot be completely excluded in the absence of immunohistochemical confirmation [33]. Nevertheless, the known mammary primary tumor, morphological similarity between the primary and metastatic lesions, and multisite distribution of disease collectively provide strong support for metastatic mammary carcinoma involving the femur.

Several limitations should be acknowledged in this case. First, the absence of computed tomography (CT) during the initial staging may have limited the detection of early osteolytic changes or other metastatic lesions that were not apparent on radiographic examination [11,20]. Second, although metastatic carcinoma was subsequently confirmed in the femoral lesion at necropsy, a bone biopsy was not performed at the time of the pathological fracture. Therefore, metastatic involvement of the femur could not be definitively established at the time of the initial fracture and was confirmed retrospectively at necropsy. Third, the temporal sequence and route of metastatic dissemination could not be determined with certainty because metastatic lesions involving the femur, regional lymph nodes, and lungs were identified at different time points. In particular, the initially negative inguinal lymph nodes and the subsequently identified popliteal and medial iliac lymph node involvement did not allow the precise pathway of metastatic spread to be established.

Overall, this case illustrates that skeletal involvement can be a clinically significant component of metastatic progression in canine mammary comedocarcinoma. The atraumatic femoral fracture and subsequent failure of fracture healing were the first clinically recognized manifestations of skeletal involvement, although the timing of metastatic colonization could not be established. Therefore, skeletal metastasis should be considered in dogs with aggressive mammary carcinoma that develop unexplained lameness, localized bone pain, progressive osteolysis, pathological fracture, or persistent failure of fracture healing.

## 4. Conclusions

This case describes extensive multisystemic metastatic progression of canine mammary comedocarcinoma with clinically significant skeletal involvement. The atraumatic pathological fracture of the femur was the first clinically recognized manifestation of metastatic disease and was followed by progressive osteolysis and failure of fracture healing despite repeated surgical stabilization. Although the chronological sequence of metastatic development could not be established, the combined clinical, radiographic, cytological, histopathological, and necropsy findings supported widespread metastatic disease. The dog died spontaneously on POD 188. Skeletal metastasis should be considered in dogs with aggressive mammary carcinoma presenting with unexplained pathological fractures or persistent failure of fracture healing, even when distant metastasis is not evident at initial staging.

## Data Availability

The original contributions presented in this study are included in the article. Further inquiries can be directed to the corresponding author.
